# Association of sleep duration with risk of all-cause mortality and poor quality of dying in oldest-old people: a community-based longitudinal study

**DOI:** 10.1186/s12877-020-01759-6

**Published:** 2020-09-21

**Authors:** Chengbei Hou, Yinan Lin, Zachary Zimmer, Lap Ah. Tse, Xianghua Fang

**Affiliations:** 1 Center for Evidence-Based Medicine, Xuanwu Hospital, Capital Medical University, Changchun Street No. 45, Xicheng District, Beijing, 100053 China; 2 Center for Applied Statistics and School of Statistics, Renmin University of China, Beijing, China; 3 School of Family Studies and Gerontology, Mount Saint Vincent University, Halifax, Nova Scotia Canada; 4 Jockey Club School of Public Health and Primary Care, The Chinese University of Hong Kong, Hong Kong, SAR China

**Keywords:** Sleep duration, Mortality, Quality of dying, Oldest old people, Penalized spline

## Abstract

**Background:**

While sleep duration has been shown to be associated with health outcomes, few studies have been conducted among the oldest old. In addition, the impact of sleep duration on quality of dying is unknown. We aimed to evaluate how sleep duration affects all-cause mortality and quality of dying in people aged 80 + .

**Methods:**

This community-based longitudinal study was performed by using data from 15,048 individuals aged ≥80 with information on sleep duration in the Chinese Longitudinal Healthy Longevity Survey. Cox and logistic regression models with penalized splines were applied to explore the shape of the association between sleep duration and all-cause mortality and poor quality of dying respectively and identify the interval of sleep duration resulting in the lowest risk of both.

**Results:**

During a median follow-up of 3.1 years, 11,582 deaths including 4116 individuals who experienced poor quality of dying were recorded. Sleep duration showed a U-shaped association with all-cause mortality and sleeping about 8 h had the minimum risk of death; a J-shaped association was found between sleep duration and poor quality of dying. Compared with sleep duration of 7–9 h, the adjusted hazard ratio of total deaths was 1.08 (95% CI 1.03–1.13) for short duration (< 7 h) and 1.12 (95% CI 1.07–1.17) for long duration (> 9 h); the adjusted odds ratio of poor QOD was 1.10 (95% CI 1.01–1.21) for long duration, but this association was restricted to those with baseline unhealthy status (P-interaction = 0.04).

**Conclusions:**

Sleeping a little longer may be better for individuals over 80 years old, and sleep duration of 7–9 h per day is optimal for both survival and good quality of life near death.

## Background

Sleep duration plays an important role in health outcomes, especially mortality [1–3]. A recent dose-response meta-analysis of prospective cohort studies reported that sleep duration of about 7 h per day is associated with the lowest risk of deaths among adults [4]. However, since sleep duration changes with age, the optimal value may vary [5]. Until now, only a few studies have investigated the relationship between sleep duration and mortality in oldest-old people, and results are mixed [6–8]. The issue of sleep amongst the very old has however broad global population health implications. Declines in fertility over the last number of decades coupled with increasing life expectancy is resulting in those 80 and older being the fastest growing age segment in the world. Determining factors that influence healthy aging among this very old population is becoming increasingly critical. Sleep has been under-appreciated as a health factor among older persons, even though the health implications of sleep may be of particular importance to those that are at high risk of mortality and chronic disease. In addition, studies that do exist almost universally consider those in developed Western countries. The population of oldest-old is however growing particularly fast in non-Western settings, including China [9]. The degree to which sleep associates with healthy longevity in these places is critical not only for population health but for assessing the universality of the sleep health association across diverse epidemiological and socioeconomic contexts.

That said, healthy longevity is recognized as being a function of not only improvements in mortality and late life health status, but also in improved quality of dying (QOD) [10–12]. Research has demonstrated that the degree of suffering and number of bedridden days near the end of life are essential dimensions of QOD [10, 13–17]. Costs of medical care have been found to be the highest at the end of life [18]. In the United States, about 25% of Medicare expenditures was spent by care for decedents in their last year of life [19]. And in Canada, more than one-fifth of healthcare spending was consumed for those in their last 6 month of life [20]. Poor QOD may therefore not only be a favorable attribute describing the end of life for people, it may be consequential in determining financial burdens within rapidly aging societies. At this time, there are no studies that examine the effect of sleep duration on QOD.

The present study addresses this gap by investigating the association of sleep duration with all-cause mortality and QOD among Chinese people aged 80 and over recruited from the Chinese Longitudinal Healthy Longevity Survey (CLHLS). The analysis uses Cox and logistic models with penalized splines, which enable examination of non-parametric associations.

## Methods

### Study design and participants

The CLHLS is a nationwide community-based survey with one of the largest samples of people in the oldest-old age group (≥80 years) in the world. The first wave was carried out in 1998, and six follow-ups with participant replacement accounting for attrition were conducted in 2000, 2002, 2005, 2008, 2011, and 2014. The sampling strategy included an attempt to interview all centenarians in selected communities, and also adopted a targeted random-sample design to ensure representativeness with approximately equal numbers of male and female nonagenarians, octogenarians, and young-old (aged 65–79 years). More details regarding study design and data quality have been described elsewhere [21, 22].

**Fig. 1:**
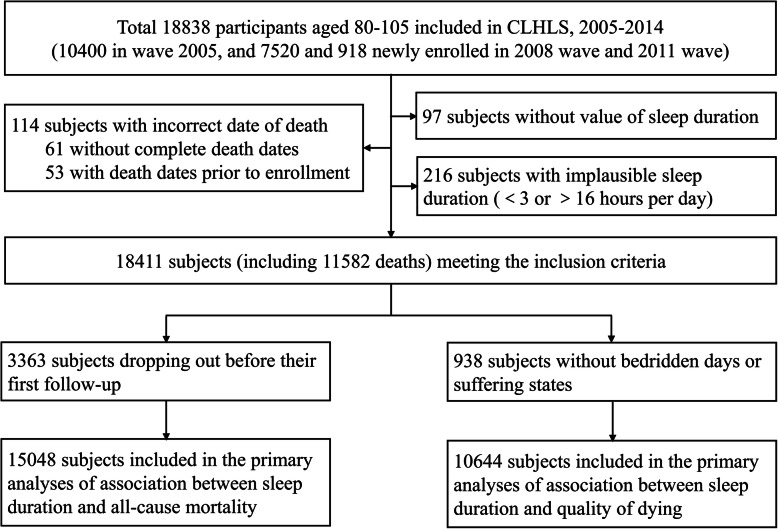
Flow chart of the study population

The current study draws on data from the last four waves 2005–2014. In the 2005 wave, 10,400 people aged 80–105 were interviewed, and 7520 and 918 were added in the 2008 and 2011 waves, respectively. We first excluded 427 participants who had incorrect death dates (*n* = 114), lacked sleep duration information (*n* = 97), or provided implausible sleep duration of < 3 or > 16 h per day (*n* = 216). This left a sample of 18,411 participants, of whom 11,582 died during follow-up. All participants are eligible for inclusion in the analysis of sleep duration and mortality. Three thousand three hundred sixty-three individuals who dropped out before a follow-up observation were however excluded, resulting in a sample size of 15,048 for this analysis. Those that died during the study period are eligible for the analysis of sleep duration and QOD. Nine hundred thirty-eight of these decedents were omitted due to the absence of information on bedridden days or suffering state, leaving a final sample of 10,644 deaths for this analysis (Fig. 1).

### Outcomes measures

Ascertainment of mortality- Date of death in the CLHLS study was obtained in multiple ways. The preferred method was obtaining dates from death certificates whenever such was available. Otherwise, dates were obtained through reports provided by next of kin or neighborhood committees. Mortality data in the CLHLS has been shown in previous analyses to be of high quality and accurate [23].

Assessment of QOD- Duration of being bedridden and degree of pain before death, as evaluated by next of kin, were adopted as components of QOD. Bedridden days before death were dichotomized into less than 30 days and 30 or more days before death, and painfulness of death was classified into “no suffering” and “suffering” [24]. Finally, we constructed two end-of-life categories by combining the two components, which were defined as “good” if a participant was bedridden for fewer than 30 days and experienced no suffering before death and as “poor” if a participant was bedridden for 30 or more days or experienced suffering before death.

### Measurement of sleep duration

The 2005 survey was the first wave to assess sleep duration. Average sleep duration per day of a participant was obtained at baseline using the question “how many hours on average do you sleep every day?” with respondents giving an integer number.

### Assessment of potential confounders

Covariates were assessed across several domains including sociodemographic characteristics (i.e., age at baseline, age at death, sex, region of residence, years of education, marital status, primary lifetime occupation, and economic condition), lifestyle (i.e., regular exercise, current smoking, and current drinking), and health factors (i.e., cognitive impairment, functional limitation, depression and chronic conditions).

Region of residence was classified as urban and rural. Marital status was dichotomized as “in marriage” if a participant was currently married with spouse present and “not in marriage” if divorced, widowed, separated or single. Primary lifetime occupation was defined according to the longest-held job during lifetime and categorized into white collar versus others. Economic condition was classified as good, fair, and poor by the question “Compared with other local people, how do you rate your economic position?”

Cognitive impairment was defined as a score lower than 18 (maximum score of 30) as assessed by the mini-mental status examination (MMSE). Functional limitation was defined as needing assistance in one or more activities of daily living (ADL) including bathing, dressing, eating, indoor transferring, toileting, and continence. Depression was defined by survey items that indicate always feeling fearful/anxious, lonely/isolated, or useless. Chronic conditions included obesity, hypertension, diabetes mellitus, cardiovascular disease, stroke, respiratory disease, and cancer. Obesity was defined as body mass index (BMI) ≥30 kg/m^2^, and the others were recorded via self-reported doctor diagnosis.

Health status was assessed at baseline and dichotomized into ‘good’ if individuals had no cognitive impairment, functional limitations, depression and reported no chronic conditions and ‘poor’ if individuals had at least one of these characteristics.

### Statistical analyses

Baseline characteristics of participants were summarized by survivorship status, and by good versus poor QOD. Cox and logistic regression models with penalized splines were used to explore the shape of the association between sleep duration and all-cause mortality and poor QOD and to identify the sleep duration with minimum risk of mortality and poor QOD after adjustment [25]. We used penalized partial likelihood to estimate parameters with sleep duration as smoothed terms. According to the corrected Akaike information criterion for smooth regression functions, a degree of freedom of 3 for sleep duration was selected [26]. 95% confidence intervals were estimated using a bootstrap method with 10,000 replicates [27]. Finally, we grouped the participants into three categories-short, recommended, and long sleep duration-based on the intersection of optimal intervals of sleep duration which had the lowest risk of all-cause mortality and poor QOD.

To quantify the associations with all-cause mortality, a Cox proportional hazards model was used to calculate hazard ratios for sleep duration as a categorical variable. In minimally adjusted models, we controlled for age at baseline and sex. Fully adjusted models included age at baseline, sex, region of residence, years of education, marital status, primary lifetime occupation, economic condition, regular exercise, current smoking, current drinking, cognitive impairment, functional limitation, depression, cardiovascular disease, stroke, respiratory disease, and cancer. The relationship between the categorical measurements for duration of sleep and risk of poor QOD was examined using a binary logistic model with similar procedures as was applied to the analyses of all-cause mortality, but adjusted for age at death instead of age at baseline. Diabetes mellitus, hypertension, and obesity might be induced by short or long sleep duration and in the causal pathway linking sleep duration with death or QOD, and therefore, were not adjusted in the primary analyses.

The proportion of missing data was 2.4% for BMI, and less than 0.5% for other covariates. We applied multivariate imputation by chained equations generating five complete datasets to deal with missing data [28]. Variables included in the primary analyses were used in imputation models. The following built-in imputation models were conducted in our analyses: for continuous variables, predictive mean matching; for binary variables, logistic regression; and for ordered categorical variables, proportional odds [29]. Final statistical inferences were obtained by pooling the separate estimates from imputed datasets according to Rubin’s rule [30].

### Sensitivity and subgroup analyses

Several sensitivity analyses were performed: (a) To examine the relation of sleep duration with mortality and QOD in all participants, we included individuals who reported the duration of sleep less than 3 h or more than 16 h per day; (b) To investigate the independent association of sleep duration with mortality and QOD, we further adjusted for diabetes mellitus, hypertension, and obesity at baseline; (c) To address the possible reverse causation between sleep duration and mortality, we omitted deaths that occurred in the first 2 years after entry; (d) To test the cut-off points of bedridden days for QOD, we used 44/45 days.

Some socioeconomic characteristics and health status might modify the association of sleep duration with mortality or QOD. Therefore, we evaluated interactions of these factors with sleep duration and conducted subgroup analyses among octogenarians, nonagenarians, and centenarians at baseline (applicable for all-cause mortality) and at death (applicable for QOD), men and women, urban and rural residents, participants in and not in marriage, participants with good, fair, and poor economic condition, and participants with good health status and poor health status.

All statistical analyses were conducted using R version 3.4.2 (R foundation for Statistical Computing).

## Results

### Participant characteristics

Table 1 presents baseline characteristics by survival status and QOD. Among 15,048 participants with complete information on survival status, the mean (SD) age at baseline was 92.8 (7.0) years, 61.6% were female, and 11,582 died during a median follow-up of 3.1 years. Among 10,644 participants with complete information on QOD, 38.7% were categorized as experiencing poor QOD.

**Table 1:** Characteristics of study population at baseline by survival status and quality of dying

Characteristics, No. (%)	Participants for sleep duration-total mortality association		Participants for sleep duration-quality of dying association	
	Alive (***n*** = 3466)	Dead (***n*** = 11,582)	Good (***n*** = 6528)	Poor (***n*** = 4116)
Female sex	2113 (60.9)	7154 (61.8)	4182 (57.7)	2683 (65.2)
Age at baseline ^a^	89.3 (6.9)	93.9 (6.7)	–	–
Age at death ^a^	–	–	96.7 (6.4)	96.5 (6.6)
Region of residence				
Urban	1357 (39.1)	4228 (36.5)	2312 (35.4)	1588 (38.6)
Rural	2109 (60.9)	7354 (63.5)	4216 (64.6)	2528 (61.4)
Educational attainment ^a^	3.1 (1.6)	1.3 (2.8)	1.3 (2.8)	1.2 (2.8)
In marriage	913 (26.3)	1651 (14.3)	945 (14.5)	558 (13.6)
White collar occupation	207 (6.0)	510 (4.4)	303 (4.6)	166 (4.0)
Economic condition				
High	595 (17.2)	1644 (14.2)	946 (14.5)	591 (14.4)
Medium	2277 (65.7)	7737 (66.8)	4378 (67.1)	2749 (66.8)
Low	594 (17.1)	2201 (19.0)	1204 (18.4)	776 (18.8)
Regular exercise	1005 (29.0)	2491 (21.5)	1514 (23.2)	768 (18.7)
Current smoking	546 (15.8)	1740 (15.0)	1066 (16.3)	541 (13.1)
Current drinking	638 (18.4)	2028 (17.5)	1196 (18.3)	669 (16.3)
Cognitive impairment	639 (18.4)	4740 (40.9)	2625 (40.2)	1770 (43.0)
Functional limitation	541 (15.6)	4152 (35.8)	2166 (33.2)	1690 (41.1)
Depression	1070 (30.9)	4552 (39.3)	2509 (38.4)	1654 (40.2)
Chronic conditions ^b^				
0	2286 (66.0)	7928 (68.5)	4546 (69.6)	2743 (66.6)
≥ 1	1180 (34.0)	3654 (31.5)	1982 (30.4)	1373 (33.4)

^a^Data reported as the mean (standard deviation) for continuous variables

^b^Included obesity, hypertension, diabetes mellitus, cardiovascular disease, stroke, respiratory disease, and cancer

### Interval of sleep duration with lowest risk of all-cause mortality and poor QOD

**Fig. 2:**
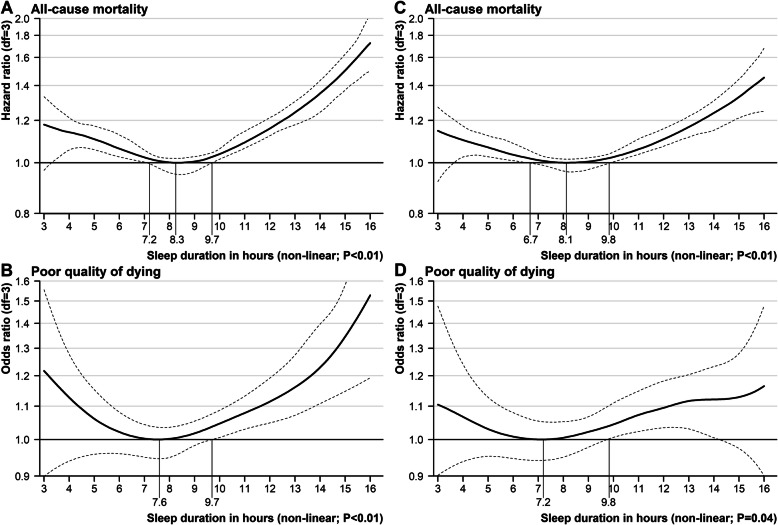
Associations of sleep duration with all-cause mortality and poor quality of dying. **a** Sleep duration-mortality association after minimal adjustment; **b** Sleep duration-poor quality of dying association after minimal adjustment; **c** Sleep duration-mortality association after full adjustment; **d** Sleep duration-poor quality of dying association after full adjustment. Solid line = hazard ratio of sleep duration (value with minimum risk as reference) for risk of all-cause mortality or odds ratio of sleep duration (value with minimum risk as reference) for risk of poor quality of dying; Dotted lines = 95% confidence interval; df, degree of freedom

There is a U-shaped association between sleep duration and all-cause mortality based on Cox models with penalized splines (Fig. 2a) and a J-shaped association between sleep duration and poor QOD based on logistic models with penalized splines (Fig. 2b). After adjusting for cofounders, participants with sleep duration of 8.1 h per day had the lowest risk of death (Fig. 2c). Compared with those sleeping 8.1 h, the risk of death was significantly higher among individuals sleeping less than 6.7 h and more than 9.8 h. Participants sleeping 7.2 h were associated with the lowest risk of poor QOD after adjustment (Fig. 2d). Compared with those sleeping 7.2 h, the risk of poor QOD was significantly higher among individuals sleeping more than 9.8 h. Sleep duration was reported as an integer number. Measured this way, the optimal interval of sleep duration with the lowest risk of all-cause mortality and poor QOD was 7–9 h. We classified sleep duration into < 7 h per day (short), 7–9 h per day (recommended), and >  9 h per day (long) for further quantitative analyses.

### Association between sleep duration and all-cause mortality

**Fig. 3:**
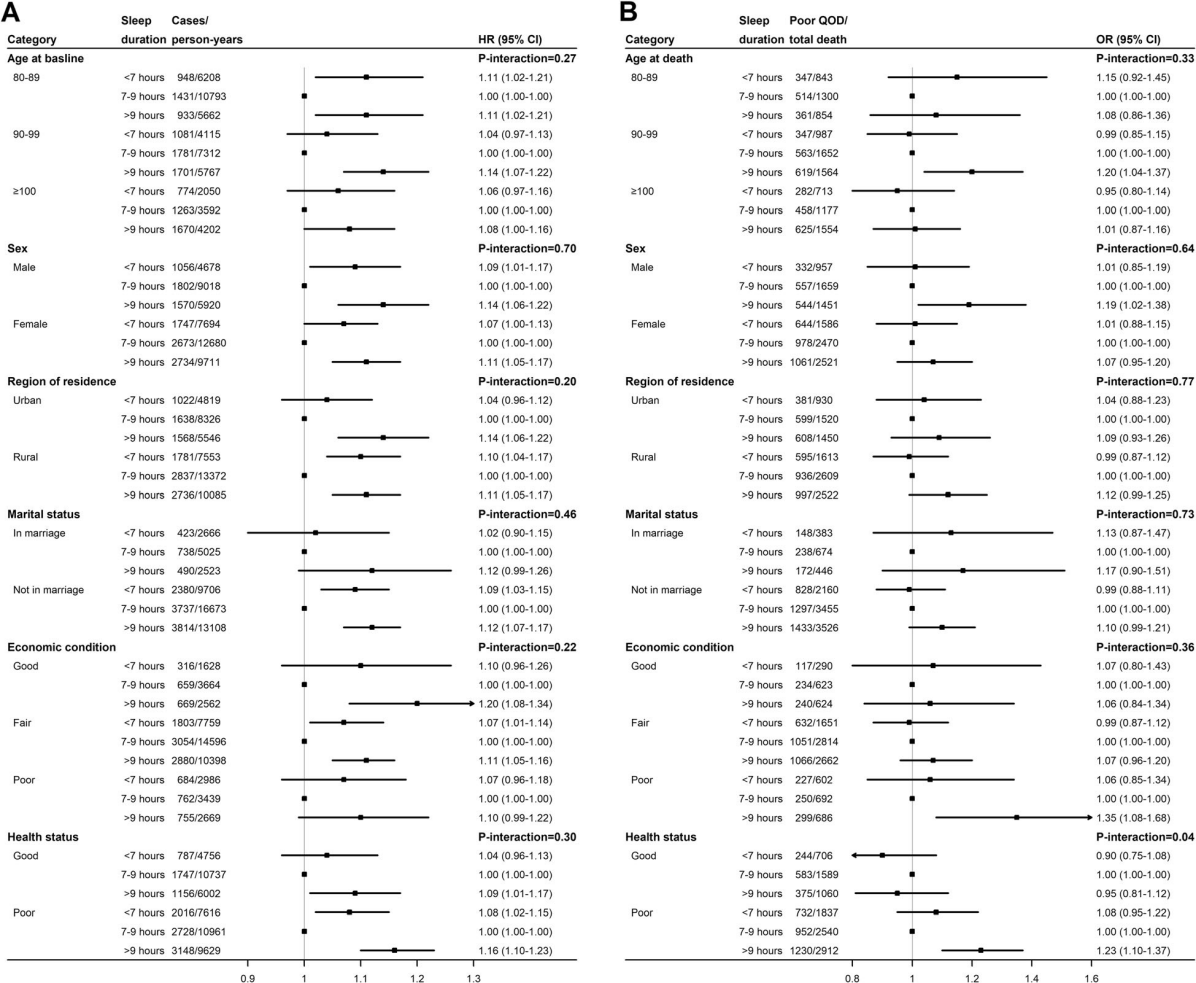
Associations of sleep duration with all-cause mortality and poor quality of dying in subgroups. **a** Sleep duration-mortality association after full adjustment; **b** Sleep duration-poor quality of dying association after full adjustment. CI, confidence interval; HR, hazard ratio; OR, odds ratio

The minimally adjusted model showed that compared with the recommended sleep duration group, both short and long sleep durations predicted a higher risk of all-cause mortality. The associations retained significance in the fully adjusted model: a hazard ratio of 1.08 (95% CI 1.03–1.13) for the short duration group and 1.12 (95% CI 1.07–1.17) for the long duration group compared with the recommended duration group (Table 2). Sensitivity analysis did not substantially alter the results. There were no significant interactions of sleep duration with baseline age group, sex, region of residence, marital status, economic condition, and health status (Fig. 3a).

**Table 2:** Hazard ratio for all-cause mortality according to categories of sleep duration

Daily sleep duration	Death cases/ person-years	Primary analyses, HR (95%CI)		Sensitivity analyses, HR (95%CI)		
		Minimally adjusted ^a^	Fully Adjusted ^b^	Model 1 ^c^	Model 2 ^d^	Model 3 ^e^
< 7 h (short)	2803/12372	1.12 (1.07–1.18)	1.08 (1.03–1.13)	1.09 (1.04–1.15)	1.07 (1.02–1.13)	1.09 (1.02–1.16)
7–9 h (recommended)	4475/21698	1.00 (Reference)	1.00 (Reference)	1.00 (Reference)	1.00 (Reference)	1.00 (Reference)
> 9 h (long)	4304/15631	1.19 (1.14–1.24)	1.12 (1.07–1.17)	1.13 (1.08–1.18)	1.11 (1.06–1.16)	1.09 (1.03–1.16)

*HR* hazard ratio, *CI* confidence interval

^a^Cox proportional hazards models were applied, with adjustment for sex and age at baseline

^b^Adjusted for age at baseline, sex, region of residence, educational attainment, marital status, primary lifetime occupation, economic condition, regular exercise, current smoking, current drinking, cognitive impairment, functional limitation, depression, cardiovascular disease, stroke, respiratory disease, and cancer

^c^Further included participants who slept less than 3 h or more than 16 h per day

^d^Further adjusted for hypertension, diabetes mellitus, and obesity at baseline

^e^Excluded deaths that occurred in the first 2 years

### Association between sleep duration and QOD

Longer sleep duration associated with a greater risk of poor QOD (Table 3). After adjustment, participants with a long sleep duration had a statistically significant 10% higher risk of experiencing poor QOD [odds ratio (OR) 1.10 (95% CI 1.01–1.21)] than those with the recommended sleep duration. Results were not different for any of the sensitivity analyses. Furthermore, there was a significant interaction effect between sleep duration and health status (*P* = 0.04); the positive association between long sleep duration and increased risk of poor QOD was found only in those with poor health status at baseline (Fig. 3b). In subgroup analyses, for participants with poor health status, the OR was 1.23 (95% CI 1.10–1.37) for the long duration group compared with the recommended duration group.

**Table 3:** Odds ratio for poor quality of dying according to categories of sleep duration

Daily sleep duration	Poor QOD/ total death	Primary analyses, OR (95%CI)		Sensitivity analyses, OR (95%CI)		
		Minimally adjusted ^a^	Fully Adjusted ^b^	Model 1 ^c^	Model 2 ^d^	Model 3 ^e^
< 7 h (short)	976/2543	1.04 (0.94–1.15)	1.01 (0.91–1.12)	1.02 (0.92–1.13)	1.01 (0.91–1.12)	1.01 (0.91–1.12)
7–9 h (recommended)	1535/4129	1.00 (Reference)	1.00 (Reference)	1.00 (Reference)	1.00 (Reference)	1.00 (Reference)
> 9 h (long)	1605/3972	1.15 (1.05–1.26)	1.10 (1.01–1.21)	1.11 (1.02–1.22)	1.10 (1.01–1.21)	1.10 (1.00–1.21)

*OR* odds ratio, *CI* confidence interval

^a^Binary logistic models were applied, with adjustment for sex and age at death

^b^Adjusted for age at death, sex, region of residence, educational attainment, marital status, primary lifetime occupation, economic condition, regular exercise, current smoking, current drinking, cognitive impairment, functional limitation, depression, cardiovascular disease, stroke, respiratory disease, and cancer

^c^Further included participants who slept less than 3 h or more than 16 h per day

^d^Further adjusted for hypertension, diabetes mellitus, and obesity at baseline

^e^Used cut-off point of 44/45 days for duration of being bedridden to define quality of dying

## Discussion

In this large community-based prospective study of oldest-old, that is people 80 and older, there was a U-shaped association between sleep duration and all-cause mortality, with those sleeping about 8 h per day having the lowest risk of death. The association between sleep duration and poor QOD was J-shaped. Given these results, sleeping between 7 and 9 h per day may be the optimal interval for both survival and good quality of life among those nearing death for individuals 80 years and older.

Consistent with our findings, a subgroup of people aged 80 + from a socio-medical survey conducted over a 10-year period in Poland demonstrated a U-shaped association between sleep duration and mortality [6]. However, that study did not conduct a quantitative analysis of death risk. Over a 20-year follow-up period, findings from the Cross-Sectional and Longitudinal Aging Study in Israel, which studied 1166 individuals between ages 75 and 94, revealed a J-shaped association, with only those whose sleep duration was longer than 9 h being at significantly higher risk of all-cause mortality compared to those that slept 7–9 h, after adjustment of multiple covariates [7]. In addition, an 8-year follow-up study of 213 people aged 80 years and older in Brazil failed to detect an association [8].

A study of 116,632 people aged 35–70 from 21 countries reported that a sleep duration of 6–8 h per day was associated with the lowest risk of death, with the optimal value to be about 7 h [31]. Our study suggests that one more hour may be advantageous for those in the oldest-old age group. Our finding was indirectly confirmed by Swedish study that indicated individuals over approximately 87 years of age who slept a little longer had a lower risk of all-cause mortality compared to their counterparts with a sleep duration of 7 h [32]. Reasons for this longer duration of sleep being more beneficial for the very old individuals are unclear and warrant future research. One possibility is that as physiological functions decline with age, and therefore sleeping for one more hour may be advantageous for restoring function and maintaining vitality.

There are biological explanations proposed for an increased risk of death with shorter sleep duration. Studies have shown that short-term sleep deprivation causes impaired glucose tolerance, higher evening cortisol levels, increased sympathetic nervous system activity, and reduced leptin secretion and elevated ghrelin [33, 34], which in turn facilitates development of diabetes mellitus, hypertension, and obesity, ultimately associating with increased mortality [35–38]. In our study effect sizes were not attenuated after adjusting for these diseases, suggesting that the relation between short sleep duration and mortality cannot be completely attributed to these factors.

Causal mechanisms for the association between long sleep duration and mortality remain unclear. One possible cause is the residual confounding of poor health status. Excessive sleep duration may be a marker of subclinical or unrecognized chronic illness [39, 40]. The severity of disease is also a determinant of the way in which health status contributes to sleep patterns [41–43]. The assessment of health status in previous studies has primarily focused on existing disease and might not sufficiently reflect individuals’ actual health conditions. In the current study, health status took into consideration a comprehensive set of indicators including chronic conditions, functional limitation, cognitive impairment, and depression. However, we did not find an interaction between sleep duration and health status, which indicated that long sleep duration might be an independent predictor of mortality.

Previous research has proposed that the measurement of QOD involves a number of dimensions. Based on earlier research, the extent of suffering and number of bedridden days are basic dimensions [13–17]. Comparisons of the current study to other research is difficult since, to our knowledge, there are no previous reports on the association between sleep duration and QOD. Our study showed a positive association between long duration of sleep and the risk of experiencing poor QOD. The association significantly varied in participants with different health statuses. This finding indicated that residual confounding (e.g. unrecognized chronic illness) might underlie the relationship.

The major strengths of our study are an unusually large sample size of people age 80+ for the examination between sleep duration and mortality and QOD; the use of a penalized spline to examine potential non-linear associations; and a methodological strategy that allowed us to identify optimal sleep duration. Moreover, this is the first investigation to examine associations between of sleep duration and both total mortality and QOD, which provided a comprehensive understanding of the relationship between sleep and healthy longevity.

This study also had several limitations. First, subjective accounts of sleep duration are likely subject to some reporting error. However, while there have been a limited number of studies that have identified lower correlations between self-reports and physiological assessments of sleep duration than is ideal, these do not indicate that biases are in any ways systematic [44]. Unfortunately, there exists very little research that allows us to assess the specific nature of reporting bias in a sample similar to the one we examine here, such as extremely old individuals with low levels of education. We might therefore extrapolate from other studies examining the association between sleep duration and mortality. These largely do not find that subjective reporting leads to difficulties in the interpretation of findings and that randomness in subjective reports of sleep duration generally result in underestimation of results [7, 31]. Although the specific sample we analyze may be unique, it is also the case that subjective reports remain the only practical and cost-effective method for large population-based surveys such as the one from which our data come and any misreporting in population-based survey methods is shared with many other exposure variables studied in lifestyle epidemiology. In the end, we suggest that there is a need for further validation of subjective reports of sleep in quality of life research among the very old, especially those in non-Western settings.

Second, due to lack of available information we were unable to adjust for sleep disorders (e.g. apnea and insomnia) which could lead to altered sleep duration and might also impact death [45, 46]. We attempted to account for sleep disorders by using BMI ≥30 kg/m^2^ as a proxy for sleep apnea [47]. Third, since we measured sleep duration only at baseline, any subsequent change in duration after recruitment could bring about non-differential misclassification affecting the estimation of the association. The use of repeated measurements of exposure through follow-up would have provided a better understanding of whether accumulation over time affects the associations between sleep duration, survival and QOD. Finally, the measure of suffering that was ascertained from close relatives of the deceased might be biased as previous research has shown that proxy reports were not always consistent with reports by individuals before death [48].

## Conclusions

Our study demonstrated that sleeping a little longer may be better for individuals over 80 years old. Sleep duration of 7–9 h per day has the lowest risk for both mortality and poor QOD. Considering shorter and longer sleepers as a high-risk population and incorporating sleep education in plans for lifestyle risk factor modification may promote longevity and decrease costs of health care for people at very old age.

## Data Availability

The datasets analyzed during the current study are available online (http://opendata.pku.edu.cn/) from Peking University Open Research Data for researchers who meet the criteria for access to these de-identified data.

## Abbreviations

CLHLS: Chinese Longitudinal Healthy Longevity Survey
QOD: Quality of dying
MMSE: Mini-mental status examination
ADL: Activities of daily living
BMI: Body mass index
SD: Standard deviation
df: Degree of freedom
HR: Hazard ratio
OR: Odds ratio
CI: Confidence interval
